# *In vitro* and *in situ* caries-preventive effect of a new combined fluoride and calcium experimental nanocomposite solution.

**DOI:** 10.1590/0103-6440202305460

**Published:** 2023-07-17

**Authors:** Karla Lorene de França Leite, Mariana Leonel Martins, Millene de Oliveira Dias, Fernanda Oliveira Miranda Tavares, Isabella Barbosa dos Santos Justino, Lúcio Mendes Cabral, Aline de Almeida Neves, Yuri Wanderley Cavalcanti, Lucianne Cople Maia

**Affiliations:** 1 Department of Pediatric Dentistry and Orthodontics, School of Dentistry, Universidade Federal do Rio de Janeiro, Rio de Janeiro, RJ, Brazil; 2 Department of Drugs and Medicines, School of Pharmacy, Universidade Federal do Rio de Janeiro, Rio de Janeiro, RJ, Brazil; 3 Department of Clinical and Social Dentistry, Universidade Federal da Paraíba, PB, Brazil.

**Keywords:** Nanotechnology, Fluorides, Calcium, Dental Biofilm

## Abstract

To assess the *in vitro* and *in situ* effect of experimental combined fluoride and calcium nanocomposite solutions on dental caries prevention. Nanocompound mesoporous silica (MS) with calcium (Ca) and sodium fluoride (NaF) - (MSCaNaF); MS with NaF (MSNaF), NaF solution (positive control), and deionized water (negative control - CG) were studied. The specimens (n=130) were submitted *in vitro* to a multispecies biofilm in the presence of 2% sucrose. After 24 h and 48 h, the culture medium pH, the percent of surface mineral loss (%SML), and lesion depth (ΔZ) were analyzed. In the *in situ* study, 10 volunteers participated in four phases of 7-days each. The products were applied on the specimens (n=240) before 20% sucrose solution drips. The polysaccharides (SEPS and IEPS), %SML and roughness (Sa) were evaluated. There was an *in vitro* decrease in pH values in 24h and 48h, compared to baseline. The MSCaNaF and MSNaF groups obtained lower values of %SML and ΔZ (p < 0.05) than CG and NaF after 24h and were similar to NaF after 48h (p<0.05). *In situ* results showed similar SEPS and IEPS among all groups after 48h. An after 7-days, the nanocomposites had similar values (p>0.05), while NaF was similar to CG (p>0.05). After 48h, the MSCaNaF and MSNaF reduced the %SML (p<0.05). After 7-days, both experimental nanocomposites were similar to NaF (p>0.05). Regarding Sa, MSCaNaF was better than NaF for both periods (p<0.05). The nanocomposites controlled the *in vitro* and *in situ* enamel demineralization, mainly in the initial periods.

## Introduction

Dental caries lesions are the result of an imbalance of de-remineralization processes occurring in the oral cavity. As a simple and low-cost treatment, professionally applied high-concentration (1.23 or 2%) topical fluoride is approved for dental caries prevention, remineralizing early enamel caries (white spot lesions) or to arrest dentine caries ^1^. In fact, fluoride-based agents have been known as a standard for caries prevention and thus, dental research is exploring other possible combinations of fluoridated compounds including calcium complexes ^2^.

In high caries-risk patients, regular visits to the dentist are necessary to keep oral health, and many previous studies have investigated the effect of various materials in dental caries prevention ^2^ ^,^ ^3^. In this regard, nanoparticulated products, with prolonged action, may keep a more effective residual effect than conventional products ^4^ ^,^ ^5^.

Mesoporous materials with high specific surface, high pore volume and unique pore size have been recently studied as biomaterials, such as carriers for controlled bioactive delivery ^6^ ^,^ ^7^. The search for new dental applications of mesoporous silica nanoparticles (MS), in the scope of the delivering active compounds, has been the focus of nanotechnology and bone tissue engineering, mainly aimed at the development of biocompatible and multifunctional nanocarriers. Previous *in vitro* studies show that calcium MS was as effective as TiF_4_and NaF to reduce erosive tooth loss ^8^ ^,^ ^9^ ^,^ ^10^ while others showed that these solutions were able to reduce enamel demineralization around orthodontic brackets ^11^. However, no study has yet compared the effect of mesoporous silica nanoparticles with added fluoride in reducing demineralization and enhancing remineralization under *in situ* cariogenic challenge. Therefore, this *in vitro* and *in situ* study was designed to identify the caries preventive effect of new sodium fluoride nanoparticle solution, with and without added calcium, in dental enamel submitted to cariogenic challenge.

## Materials and Methods

### Preparation of the Experimental Nanocomposites

Mesoporous silica (MS) based nanocomposites were obtained by a nanoprecipitation technique, varying the molar ratio of water/tetraethoxysilane (TEOS), NH3/TEOS and the amount of cetyltrimethylammonium bromide. All characterization analyses are described as previously reported in the literature ^11^.

Thereafter, a sodium fluoride solution (Aldrich Chemical Co^®^, Saint Louis, USA) was included, to which calcium (Ca) was added or not. The following experimental solutions were produced: 1) an experimental nanocomposite of mesoporous silica (MS) dopped with calcium and sodium fluoride (MSCaNaF) and 2) MS with NaF (MSNaF). The nanocomposites were analyzed by ICP-AES, prepared by a surfactant templated, base-catalyzed condensation procedure with Ca(NO_3_)_2_ added in the parent solution as a calcium precursor (in order to obtain the calcium concentration) and by ion chromatography with conductivity detection to obtain the fluoride concentration. ICP-AES results were 10.7 ± 0, 8% [w /w] of medium content. The determination of fluoride resulted in 9.3 ± 0.1% [w /w] of medium content ^11^.

### In vitro study

### Study Design and Sample Size

The *in vitro*, randomized, controlled, single-blind study was based on a previous investigation that evaluated the enamel mineral loss reduction resulted from application of nanocomposite solutions containing calcium and fluoride that used a specific sample size (n=13 per group) ^(^ ^11^. A 0.8 statistical power was used to detect a 50% significant difference in mean mineral loss in each treatment group compared to the control group (1.36% of NaF, 6135 ppm of F^-^), using a one-tailed test with a 5% significance level (BioEstat 5.3^®^, Instituto de Desenvolvimento Sustentável Mamirauá, Tefé, Brazil).

### Specimen Preparation

Enamel specimens (4×4×4 mm^3^) were prepared from bovine crowns as described previously ^12^. Surface hardness was determined on the enamel specimens ^13^ and those in the ±10% range were selected, according to the total mean of the baseline microhardness (320.76 kgF/mm^2^) and were randomized among the groups. From these groups, 130 sound enamel blocks were selected for the *in vitro* study. After that, half of the specimens’ surface were covered with an acid-resistant nail varnish in order to create an unexposed area (sound area) and one exposed area.

After random distribution of the specimens (Microsoft Excel^®^) in each of the groups (n=13), the specimens were transferred to a 12-well polystyrene plate (K12-024, Kasvi^®^, São José do Pinhal, Brazil), and sterilized under ultraviolet light (40W) for 1 h ^14^ ^,^ ^15^.

A single blinded trained researcher actively applied the test products (100μL) in the exposed area using a microbrush (KG Sorensen^®^, Cotia, Brazil) for 1 min on each enamel block before the cariogenic challenge.

### Cariogenic Challenge

After reactivation of *Streptococcus mutans* (ATCC 25175), *Streptococcus salivarius* (ATCC 7073), *Streptococcus sanguinis* (ATCC 20556), and *Lactobacillus casei* (ATCC 393) strains, a bacterial suspension was prepared according to CLSI (2012) ^16^ ^)^ standards and transferred to BHI broth containing 2% sucrose (pH=7.10). Previously treated specimens were immersed in artificial saliva for 1 h ^17^. After, 5 mL of the mixed inoculum (5 x 10^5^ CFU/mL of the final concentration) was added, and the specimens were incubated at 37 °C for 24 h and 48 h.

The growth control (GC) specimens contained a bacterial suspension (multispecies biofilm of *Streptococcus* spp. and *Lactobacillus casei*) prepared in BHI broth with 2% sucrose, while the sterility control (SC) specimens included BHI broth with 2% sucrose and both did not receive the experimental treatments. The following groups were produced: MSCaNaF and MSNaF (experimental groups), NaF (positive control), GC (negative control) and SC (sterility control).

### Data Collection and Analysis

After 24h (n=65) and 48h (n=65), the specimens were sonicated for 1 min and the acidogenicity of culture medium was assessed by pH measurements (PHOX^®^, Colombo, Brazil). The procedure was carried out in duplicate by a blinded trained examiner.

All enamel blocks of each group were reassessed after the cariogenic challenge by the same examiner to determine the final surface microhardness, according to the parameters established for the baseline assessment. The percent loss of surface mineral (%SML) was obtained after the experiment ^13^.

For the analysis of mineral content in the lesion, randomly selected enamel blocks (n= 6, per group) were scanned on a high-energy micro-CT scanner (Skyscan 1173, Bruker, Kontich, Belgium) using the following acquisition parameters: 70 kVp, 114 mA, pixel size of 7.12 μm, and 1mm aluminum filter. The images were later reconstructed into cross sections using a dedicated software (NRecon, Bruker) and specific reconstruction parameters. The integrated mineral loss (∆Z) was calculated by drawing a profile across the enamel surface and measuring the integrated area under the curve, corresponding to the mineral density of the carious lesion ^11^.

### In situ study

### Ethical Aspects and Sample Size

The *in situ* protocol was approved by the Research Ethics Committee (protocol No. 2.996.144/2019). The *in situ* study was based on the findings of the *in vitro* study (pilot of the study), which found that appropriate caries lesions were obtained in a 24h and 48h period of cariogenic challenge. Thus, the participants were requested to use additional extraoral sucrose dipping to enhance the caries process for the period of 24h and 7 days for this *in situ* study.

The obtained data were used to perform a sample size calculation for paired differences based on the reduction in enamel mineral loss. The calculation considered the mean difference between pairs and the standard deviation of the differences. Based on these data, we assumed that the study would require a power of 80% and a level of significance of 5%, and as a result, six blocks of bovine enamel were required per volunteer in each phase, with a minimum of nine volunteers. Considering 10% loss of volunteers, the present study was carried out with 12 volunteers.

Prior to enrolling into the study, an independent examiner, not otherwise involved in the study, conducted a clinical examination to assess caries status and to determine any treatment needs of the potential volunteers. These were undergraduate and postgraduate dental students, who fulfilled the inclusion criteria (salivary flow rate > 1mL/min, good general and oral health with no active caries lesions or periodontal treatment needs, ability to comply with the experimental protocol, no antibiotic use during the 1 month prior to the study, use of any form of medication that modifies salivary secretion, not using a fixed or removable orthodontic device) and consented to participate ^18^. The mean age of the subjects was 23.1 ± 4.0 years old; the mean colony forming units (CFU) count was 9.2 ± 2.3 (Log_10_) and the mean ICDAS index was 2.3 ± 3.1.

### Study Design

**Figure 1:**
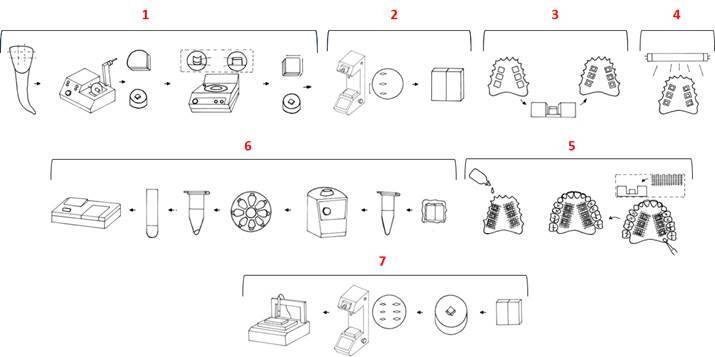
Study design: 1- Specimen preparation; 2- Baseline microhardness; 3- Sample randomization; 4- Sterilization of the appliances; 5- Experimental protocol; 6- Analysis of the dental biofilm; 7- Microhardness analysis; 8- Surface topography analysis.

The study had an *in situ*, triple-blind (by operator and volunteers regarding product use and by the examiner assessing the outcomes), crossover design and was conducted in four experimental phases of seven days each. A minimum of 48 hours was considered as washout periods. The subjects used palatal appliances containing three sound enamel specimens on each side, with predetermined initial surface hardness. In each phase, groups of volunteers were subjected to one of the following treatments: MSCaNaF, MSNaF, NaF (positive control) and negative control (deionized water) applied once on each specimen at the beginning of the experimental phase. After the first 24h of appliance use, sucrose was dropped three times per day on sound specimens to simulate a cariogenic challenge. At the end of each phase, the concentration of soluble and insoluble extracellular polysaccharides (SEPS and IEPS) in the biofilm was assessed. The caries preventive effect of each treatment was evaluated by surface hardness. Also, volumetric roughness (Sa) was used to assess enamel topography. For all analyses, the samples were blindly analyzed using codes (Figure 1).

### Specimens Preparation

Enamel specimens (4×4×2 mm) were prepared from bovine incisor crowns, as described previously ^12^. After baseline microhardness measurements (314.42 kgF/mm^2^) the specimens were randomized across the groups. From these groups, 240 sound enamel blocks were selected for the *in situ* study. An unexposed area (sound area) and the exposed area were also created on the specimens (Figure 1).

### Palatal Appliance Preparation

Six specimens were randomly attached in each acrylic resin palatal appliance, with three enamel blocks on each side of the appliance (Figure 1). The random sequence of specimens started from the anterior to posterior. This appliance setup was fixed for each volunteer at the different phases. A plastic mesh was fixed 1 mm above the dental specimens to favor biofilm accumulation^12^. After, the appliance was sterilized under ultraviolet light (40 W) (t = 1h) ^14^ ^,^ ^15^.

### Experimental Protocol

The sequence of experimental protocol followed by the volunteers in each phase was randomized in blocks of twelve, generated using https://www.random.org/. The randomization list was kept blinded by one researcher (L.C.M) and this list remained secured until the completion of all data collection in the main study. Thus, in each phase, all test products were used by groups of volunteers such as at the end of the experiment, all volunteers experienced all products. The crossover study was conducted in four phases of seven days each, in which the volunteers were randomly allocated to the following test products: MSCaNaF, MSNaF, NaF, and control group (CG) (deionized water).

At the beginning of each phase, the volunteers placed the device in the mouth for 5 min to allow saliva pellicle formation. After that, the appliances were removed and a blinded researcher (K.L.F.L) applied the test products (100 μL) using a micropipette for 1 min on each enamel block, before the cariogenic challenge.

The volunteers used the appliances for 24 h and after this period, they were instructed to drop a 20% sucrose solution onto the specimens three times a day, with the appliance outside the mouth. The sucrose solution was allowed to rest onto each enamel block for 5 min. After 48 h, three enamel blocks were removed from the same side of appliance. The same procedure was performed for the other side at the end of the experimental protocol (7 days). Washout periods of at least 48 hours were established between each phase.

During the experiment and in the washout periods, the volunteers brushed their teeth and the appliance outside the mouth (except for the area containing the specimens) with a fluoridated toothpaste (Oral B, Procter & Gamble^®^, USA) and were instructed not to use mouth rinses. Considering the crossover design of this study, no restrictions were made regarding the volunteer’s diet. The volunteers used the appliances throughout the whole experimental phase, removing them only during the sucrose treatment, during food consumption, beverage intake and during oral hygiene procedures.

### Data Collection and Analysis

After each experimental phase, the biofilms formed in each specimen were collected. This sample was used to evaluate the concentration of soluble (SEPS) and insoluble (IEPS) extracellular polysaccharides. To the collected biofilm, 1 mL of 0.9% NaCl solution was added in each microtube and, after being vortexed, the suspension was centrifuged at 3000 *g* for 10 min. An aliquot of 500 μL of the sonicated biofilm suspension was used for extraction of polysaccharides, as described previously ^19^. The total amount of carbohydrates in each sample was quantified by the phenol sulfuric method with glucose as standard ^20^ ^,^ ^21^. Samples were analyzed in a spectrophotometer (490 nm) and the absorbance values were interpolated in a standard curve with known concentrations (μg/mL) of glucose.

After data collection for SEPS and IEPS, the surface microhardness of the enamel blocks was measured in all groups in the same way as performed initially and after, the %SML was calculated.

The surface roughness of the samples was measured by 3D non-contact profilometry (Nanovea PS50 Optical, Nanovea, Irvine, USA). A 1 mm^2^ assessment area on the enamel blocks were standardized. A chromatic confocal sensor using a white light axial source, with a scan velocity of 2 mm/s and a refraction index of 10,000 were used to capture 3D images. The means for the three volumetric roughness (Sa) (ISO 25178) (250 μm^2^) measurements were obtained for each specimen ^22^.

### Statistical Analysis

The normal distribution of data was tested for all variables using the Shapiro-Wilk test. For the *in vitro* study, Wilcoxon, Kruskal Wallis and Mann Whitney tests (p < 0.05) were considered for the pH, %SML and ΔZ considering the periods of 24 h and 48 h. For the *in situ* study, data that did not satisfy assumptions of equality of variances and thus, normal distribution of errors were transformed. The data were independently analyzed at each period and between them (48 h and 7 days). Analysis of Variance (ANOVA) checked the effect of the treatments with repeated measures, with Tukey and Bonferroni post-hoc tests, considering p < 0.05, and volunteers were considered as statistical blocks in those analyses.

## Results

**Figure 2:**
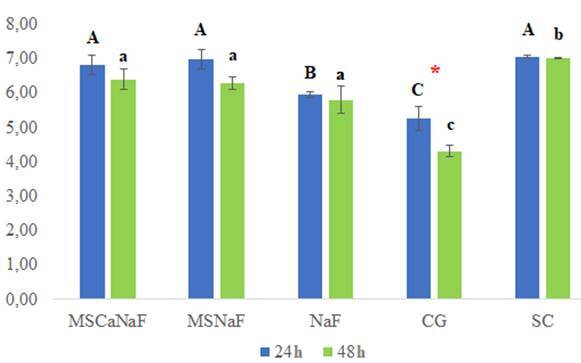
pH values after 24 h and 48 h of biofilm formation for each experimental group.

For the *in vitro* study, pH measurements were different after 24h and 48h for all groups, except for the SC group (Figure 2). No difference in the MSCaNaF and MSNaF groups was seen after 24h (p<0.05), but after 48h, pH was similar (p>0.05).

**Figure 3:**
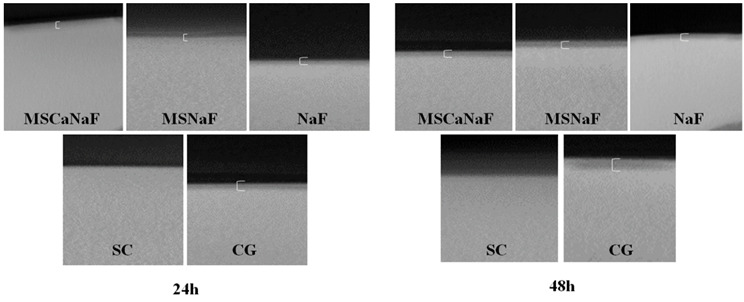
Photomicrograph of enamel surface assessed by micro-CT in the *in vitro* study. The delimited region indicates the area and depth of the carious lesion, from where ΔZ values were obtained.

The MSCaNaF and MSNaF groups resulted in lower % SML and ΔZ (p<0.05) than CG and NaF after 24h. However, after 48h these were similar to NaF (p>0.05) and different from CG (p<0.05) (Table 1 and 2 and Figure 3).

**Table 1:** Surface microhardness analysis before and after the experiments and percentage of enamel surface microhardness loss for the *in vitro* study.

Groups	24 h			48 h		
	SMH Before	SMH After	%SML	SMH Before	SMH After	%SML
MSCaNaF	328.29 ± 5.17^Aa^	259.65 ± 13.63^Ba^	8.64 ± 2.48 ^a^	329.64 ± 2.19^Aa^	205.57 ± 24.72^Ba^	27.28 ± 7.75^a^
MSNaF	339.01 ± 3.77^Aa^	262.95 ± 18.09^Ba^	6.06 ± 5.76 ^a^	320.26 ± 3.14^Aa^	194.20 ± 17.81^Ba^	28.82 ± 9.39^a^
NaF	339.88 ± 3.66^Aa^	210.07 ± 20.75^Bb^	15.60 ± 6.66^b^	325.55 ± 3.38^Aa^	185.07 ± 25.74^Ba^	37.52 ± 5.32 ^a^
CG	336.25 ± 2.45^Aa^	178.63 ± 16.99^Bc^	36.87 ± 10.57^c^	334.11 ± 5.50^Aa^	94.35 ± 24.39^Bb^	69.81 ± 7.00^b^
SC	327.69 ± 4.33^Aa^	306.86 ± 10.87^Bd^	3.39 ± 3.13^d^	330.14 ± 4.94^Aa^	301.54 ± 13.91^Bc^	3.58 ± 4.20^c^

Means followed by different letters are statistically different (p < 0.05). Uppercase letters show differences before and after the experiment in each group (Paired Samples t Test, p < 0.05) and lowercase letters in the same column show differences between the treatments (Kruskal-Wallis and Mann-Whitney; p < 0.05).

**Table 2:** Mineral loss analysis (∆Z) for the *in vitro* study.

Groups	∆Z (8-bit gray values)		
	24 h	48 h
MSCaNaF	37.15 ± 9.88 ^Aa^	67.65 ± 7.45 ^Ba^
MSNaF	41.32 ± 7.81 ^Aa^	71.87 ± 12.11 ^Ba^
NaF	55.71 ± 8.13 ^Ab^	80.15 ± 7.89 ^Ba^
CG	131.23 ± 16.43 ^Ac^	217.19 ± 22.65 ^Bb^
SC	22.95 ± 6.34 ^Ad^	26.62 ± 8.25 ^Ac^

Means followed by different letters are statistically different (p < 0.05). Uppercase letters show differences before and after the experiment in each group (Paired Samples t Test, p < 0.05) and lowercase letters in the same column show differences between the treatments (Kruskal-Wallis and Mann-Whitney; p < 0.05).

For the *in situ* study, two volunteers were excluded due to the use of antibiotics. Therefore, this study was carried out with two 240 enamel blocks distributed in 10 volunteers. No adverse effects were observed in any of the intervention groups. The amount of SEPS and IEPS increased between periods of 48h to 7 days for all groups (p<0.05). In the 48h period, values of SEPS and IEPS were similar for all groups, however, after the 7 day period, SEPS values of the nanocomposites were similar (p>0.05) but differed from CG (p<0.05), while NaF was similar to CG (p>0.05). All experimental groups had similar values of IEPS (p>0.05) which were lower than those for the CG (p<0.05) (Table 3).

In relation to surface microhardness, significant intragroup differences in the %SML were found for the evaluated periods. After 48h, MSCaNaF and MSNaF groups were more effective compared with NaF in reducing demineralization, with the lowest %SML observed (p<0.05). However, after 7 days, the MSCaNaF, MSNaF and NaF groups were similar (p>0.05) (Table 3).

**Figure 4:**
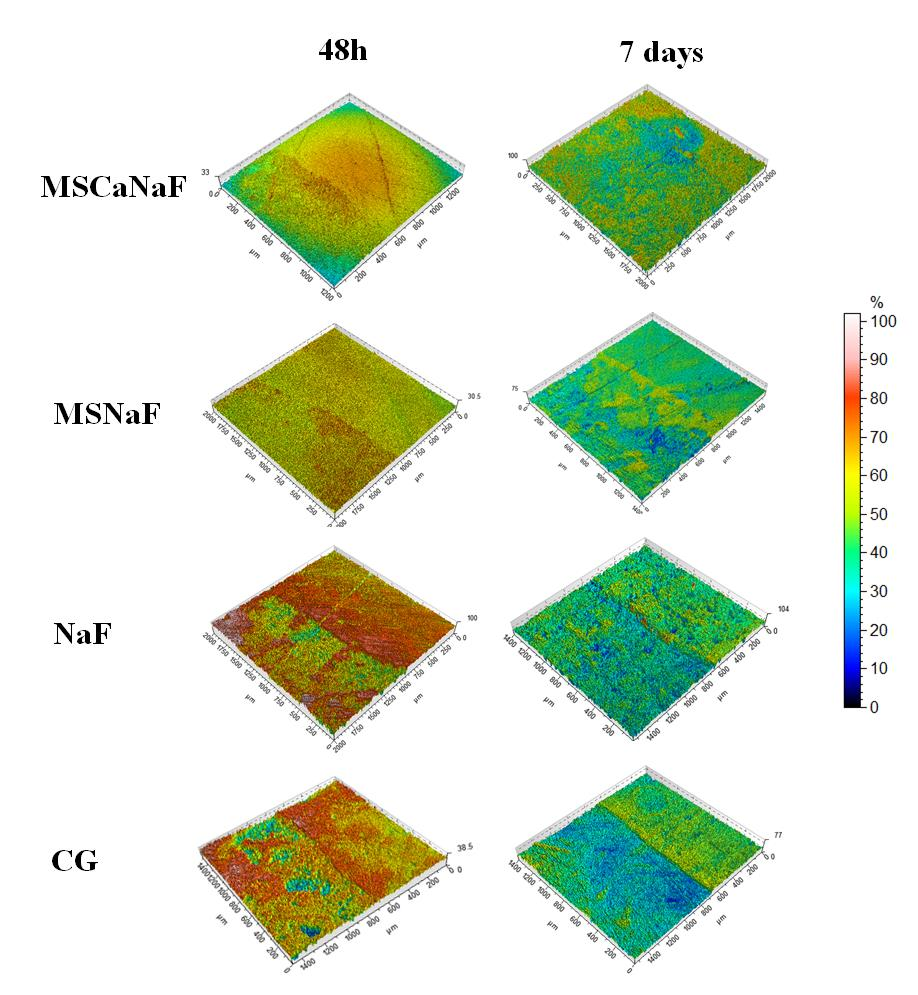
Photomicrographs of the surface of the enamel blocks after the caries challenge obtained via non-contact profilometry. The left side of the image represents the Sa eroded.t

Regarding roughness, the 7 days values were higher than the 48 h values for all groups (p<0.05). The greatest inhibition of tooth structure loss occurred when specimens were treated with MSCaNaF for both analyzed periods (p<0.05) (Table 4). The solutions prevented greater surface changes generated by acids from bacterial metabolism for all groups. The highest roughness values were observed in the CG (Figure 4).

**Table 3:** Mean and standard deviation of the concentration of soluble (SEPS) and insoluble (IEPS) extracellular polysaccharides and enamel surface microhardness analysis, according to the periods of 48 h and 7 days.

Groups	SEPS		IEPS		% SML	
	48 h	7 days	48 h	7 days	48 h	7 days
MSCaNaF	9.65 ^Aa^ ± 14.28	25.67 ^Ab^ ± 34.83	5.14 ^Aa^ ± 18.48	51.87 ^Ab^ ± 24.06	15.65 ^Aa^ ± 7.86	32.11 ^Ab^ ± 6.86
MSNaF	9.26 ^Aa^ ± 14.19	33.44 ^ABb^ ± 29.78	7.64 ^Aa^ ± 14.63	64.92 ^Ab^ ± 26.95	18.67 ^Aa^ ± 5.94	32.32 ^Ab^ ± 7.74
NaF	16.87 ^Aa^ ± 15.81	50.66 ^BCb^ ± 44.72	17.44 ^Aa^ ± 19.80	73.55 ^Ab^ ± 27.32	23.90 ^Ba^ ± 8.39	33.72 ^ABb^ ± 11.77
CG	15.69 ^Aa^ ± 44.92	81.10 ^Cb^ ± 48.87	24.13 ^Aa^ ± 14.31	106.16 ^Bb^ ± 40.89	33.88 ^Ca^ ± 6.67	45.43 ^Bb^ ± 14.96

Different uppercase letters in the same column mean intergroup statistically significant difference, and different lowercase letters represent statistical differences between the periods (p < 0.05).

**Table 4:** Mean and standard deviation of surface enamel obtained via non-contact profilometry for Sa of groups after caries challenge.

Groups	Sa sound		Sa caries challenge	
	48 h	7 days	48 h	7 days
MSCaNaF	0.62 ^Aa^ ± 0.22	0.62 ^Aa^ ± 0.27	1.14 ^Ab^ ± 0.34	1.63 ^Ac^ ± 0.41
MSNaF	0.49 ^Aa^ ± 0.23	0.53 ^Aa^ ± 0.15	1.33 ^ABb^ ± 0.38	1.88 ^ABc^ ± 0.36
NaF	0.54 ^Aa^ ± 0.32	0.48 ^Aa^ ± 0.27	1.67 ^Bb^ ± 0.31	2.10 ^Bc^ ± 0.37
CG	0.60 ^Aa^ ± 0.22	0.63 ^Aa^ ± 0.19	2.23 ^Cb^ ± 0.48	3.60 ^Cc^ ± 0.95

Different uppercase letters in the same column mean intergroup statistically significant difference, and different lowercase letters represent statistical differences between the periods (p < 0.05).

## Discussion

As seen in the present *in vitro* and *in situ* study, the use of MS-nanoparticles associated with fluoride and calcium is effective for dental caries prevention. Mesoporous silica nanoparticles have indeed attracted considerable attention for their application in drug delivery and biomedicine, due to the large surface area and pore volume ^4^ ^,^ ^5^ ^,^ ^23^, and thus, studies with new preventive products are welcome in dental practice.

Several studies report the recent advances in MS-nanoparticles, including immediate and sustained delivery systems, as well as controlled release and targeted drug delivery systems ^24^ ^,^ ^25^. In dentistry, the development of nanohydroxyapatite with MS for therapeutic management of dentin surfaces ^26^ and the antibacterial dental composites with chlorhexidine-based MS ^27^ ^,^ ^28^ have been reported. However, no previous results on the action of these proposed new mesoporous silica nanoparticles with fluoride products in the enamel were found.

Our *in vitro* model showed that MSCaNaF and MSNaF were effective in reducing enamel demineralization (% SML and ΔZ). Even though no statistically significant difference was found between nanocomposites and NaF after 48 h, enamel samples treated with nanocomposites presented a higher protective effect compared with NaF in 24h. Therefore, the MSCaNaF and MSNaF groups may be considered promising alternatives in the clinical control of dental caries since results were also similar in the *in situ* study. However, in both *in vitro* and *in situ* studies, their long-term maintenance of F levels has reduced over time. In the present study, these products were applied only once before the cariogenic challenge, in an attempt to evaluate their preventive effect. The *in situ* study was carried out over a longer period, and nevertheless, in the period of 7 days, these products were still able to maintain the same levels of demineralization compared to NaF. Possibly, the nanocomposites are more effective at the beginning of the demineralization process (24h and 48h period).

Although the amount of SEPS and IEPS in the 48h period was the same for all products at the end of the cariogenic challenge, all test products showed higher values of both SEPS and IEPS. This increase in the polysaccharide values can be considered as a possible explanation for the caries progression in all groups over the experimental period. However, the authors emphasize that this increase was higher in the control group compared to the experimental, meaning that all fluoride products were able to reduce the progression of dental caries lesions formation.

In general, nanocomposites of MSCaNaF and MSNaF showed better results compared with NaF, and this may be due to the nanocomposite particles with a high silica encapsulation efficiency. It seems there was no synergism between calcium and fluoride since there was no statistical difference between the nanoparticles. In the present study, the authors used the products in solution form, which may have resulted in low fluoride retention in the dental enamel, and this hindered the maintenance of the preventive effect during the 7 days. Other formulations, such as varnishes, present greater retentivity, and, probably, a greater amount of fluoride is released over a long-term^29^. It is possible that the effectiveness of the nanocomposites was compromised due to their presentation, since solutions have less adhesion on the dental surface than other formulations, such as varnishes and gels ^30^. Despite this, the solutions from the present study managed to release loosely bound fluoride that may be an important source of fluoride on the enamel surface to induce remineralization and reduce demineralization during periods of cariogenic challenge.

Regarding the enamel morphological characteristics, the groups treated with MS-nanocomposites showed a decrease in surface roughness (Sa), mainly after 48h. High concentrations of NaF can be a physical barrier, inhibiting contact of the acid with the dental surface and/or acting as a fluoride reservoir since it is able to promote the precipitation of CaF_2_ ^31^. However, although the NaF group has equal fluoride concentration as the nanocomposites, different results were observed. It could be justified by the gradual F^-^ release of the nanocomposites, extending the effect of these products. The enamel morphological characteristic is corroborated by the photomicrographs of the surface of the enamel specimens, where it is possible to observe larger exposure of enamel prisms in the NaF and control groups. After 7 days, the photomicrographs showed that the MSCaNaF and MSNaF could not completely protect the enamel surface during all cariogenic challenges.

As the present study was the first to be carried out in an *in vitro* and *in situ* model, more clinical studies are desirable to identify the effects of these experimental nanoparticles, against dental caries, with other presentation forms and different concentrations, for a long time and with periodic exposure.

## Conclusion

MSCaNaF and MSNaF were able to decrease the enamel demineralization, mainly in the initial periods evaluated. Although there was a reduction in the efficacy of the nanocomposite products over the *in vitro* and *in situ* experimental period, they were similar to sodium fluoride and superior to negative control for all parameters analyzed.
